# Synthesis and Characterization of Bioactive Acylpyrazolone Sulfanilamides and Their Transition Metal Complexes: Single Crystal Structure of 4-Benzoyl-3-methyl-1-phenyl-2-pyrazolin-5-one Sulfanilamide

**DOI:** 10.1155/2015/717089

**Published:** 2015-05-27

**Authors:** Omoruyi G. Idemudia, Alexander P. Sadimenko, Anthony J. Afolayan, Eric C. Hosten

**Affiliations:** ^1^Chemistry Department, University of Fort Hare, Private Bag X1314, Alice 5700, South Africa; ^2^Botany Department, University of Fort Hare, Private Bag X1314, Alice 5700, South Africa; ^3^Chemistry Department, Nelson Mandela Metropolitan University, P.O. Box 77000, Port Elizabeth 6031, South Africa

## Abstract

Two Schiff base ligands Ampp-Sn** 1** and Bmpp-Sn** 2**, afforded by a condensation reaction between sulfanilamide and the respective acylpyrazolone carbonyl precursors, their Mn(II), Co(II), Ni(II), and Cu(II) complexes prepared by the reaction of ligands and corresponding metal salts in aqueous solutions, were synthesized and then characterized by both analytical and spectroscopic methods, in a view to developing new improved bioactive materials with novel properties. On the basis of elemental analysis, spectroscopic and TGA results, transition metal complexes, with octahedral geometry having two molecules of the bidentate keto-imine ligand each, have been proposed. The single crystal structure of Bmpp-Sn according to X-ray crystallography showed a keto-imine tautomer type of Schiff base, having three intramolecular bonds, one short N2⋯H2⋯O3 hydrogen bond of 1.90 Å and two long C13⋯H13⋯O2 and C32⋯H32⋯O3 hydrogen bonds of 2.48 Å. A moderate to low biological activities have been exhibited by synthesized compounds when compared with standard antimicrobial agents on screening the synthesized compounds against* Staphylococcus aureus*,* Bacillus pumilus*,* Proteus vulgaris*, and* Aeromonas hydrophila* for antibacterial activity and against free radical 1, 1-diphenyl-2-picryl-hydrazyl (DPPH) for antioxidant activity.

## 1. Introduction 

The electrophilic carbonyl carbon in aldehydes and ketones is a target site for nucleophilic primary amines to form an interesting group of ligands known as Schiff bases* via* a condensation reaction. Schiff bases possess remarkable reactivity and properties which have been observed to be more efficient when in coordination with transition metals, hence their diverse applications [1–8]. Acylpyrazolones are a series of heterocyclic diketones that have attracted a lot of research attention because of their chemistry and strong chelating properties especially towards transition metal ions [9] and numerous uses [10]. Knorr first synthesized pyrazolone derivatives by reacting phenyl hydrazine with ethylacetoacetate to form a novel 1-phenyl-3-methyl-5-pyrazolone in 1883 and studies on this group of compounds have been on since then [11]. They have the ability to exist either in an enol or keto tautomer forms, which gives them the potential to form different types of interesting coordination compounds [12, 13]. Sulfanilamides are the simplest of the family of sulfa drugs. They act as metabolites capable of obstructing folic acid synthesis in bacteria, thereby causing cell death, the reason for their strong antibacterial activity [14]. There has been hypothesis that some metal complexes of sulphur containing ligands have shown more potency in their anticancer properties [15, 16]. Coombs and coworkers synthesized Schiff bases of sulfanilamide with salicylaldehyde and their palladium complexes [14]. The single crystal structure of another salicylaldehyde Schiff base, N, N′-(*p*-phenyl sulfanilamide)-5,6-dimethoxypyrimidine-(2-hydroxyl benzene) methylidene, with excellent fluorescent properties has also been resolved [17]. We have reported the X-ray crystal and molecular structures of 4-benzoyl derivatives Schiff bases, 4-benzoyl-3-methyl-1-phenyl-2-pyrazolin-5-one phenylhydrazone [18] and 4-benzoyl-3-methyl-1-phenyl-2-pyrazolin-5-one-2,4-dinitrophenylhydrazone [19]. In continuation of our probe on new Schiff bases and taking the advantage of possible synergistic properties from the combination of acylpyrazolone and sulfanilamide, presented herein are the synthesis, characterization, antibacterial and antioxidant studies of 4-acylpyrazone with 4-aminobenzenesulfonamide Schiff bases and their Mn(II), Co(II), Ni(II), and Cu(II) complexes.

## 2. Experimental

### 2.1. Materials and Methods

Melting point was determined using the GallenKamp melting point apparatus. CHN elemental analyses were carried out on a LECO.TRUSpec Micro CHNS analyzer. FTIR spectra were measured as KBr Pellets (4000–370 cm^−1^) on a Perkin-Elmer Model System 2000 FTIR spectrophotometer. Electronic spectra of metal complexes were recorded on a Perkin-Elmer Lambda 25 spectrophotometer and a Sherwood Scientific magnetic susceptibility balance was used for magnetic moments with powder samples of complexes at room temperature. Diamagnetism corrections were estimated from Pascal's constants. ^1^H and ^13^C NMR spectra in deuterated DMSO were recorded on a Bruker 600 MHz Avance II NMR spectrophotometer using trimethylsilane TMS, as internal standard. Chemical shifts are given in ppm (*δ* scale). Thermal analyses were done on a NETZSCH STA 449 C instrument at a temperature range of 20–900°C with a heating rate of 20°C min^−1^ in nitrogen gas. Mass spectra of ligands were determined by the Bruker micrOTOF-Q II 10390 mass spectrometer. They were analyzed with ACPI using a direct insertion probe (DIP). An external calibration with sodium formate was performed to attain the correct accurate mass. Single crystal X-ray diffraction studies were performed at 200 K using a Bruker Kappa Apex II diffractometer with graphite monochromated Mo K*α* radiation (*λ* = 0.71073 Å). All reagents, Manganese(II) chloride tetrahydrate MnCl_2_·4H_2_O, Cobalt(II) chloride hexahydrate CoCl_2_·6H_2_O, Nickel(II) chloride hexahydrate NiCl_2_·6H_2_O, Copper(II) acetate monohydrate (CH_3_COO)_2_Cu·H_2_O, sulfanilamide, and 3-methyl-1-phenyl-2-pyrazolin-5-one, and solvents were of analytical grade as supplied by Aldrich. Schiff base precursors 4-acyl-3-methyl-1-phenyl-2-pyrazolin-5-one derivatives were synthesized and purified as reported earlier [20].

### 2.2. General Procedure for the Synthesis of Acylpyrazolone Schiff Bases

To a solution of 4-benzoyl-3-methyl-1-phenyl-2-pyrazolin-5-one (2.0 mmol, 0.56 g) and 4-acetyl-3-methyl-1-phenyl-2-pyrazolin-5-one (2.0 mmol, 0.43 g) in methanol (40 mL) each in a separate round bottom flask sulfanilamide was added in drops (2.0 mmol, 0.34 g) in hot methanol while stirring; Bmpp-Sn and Ampp-Sn were precipitated, respectively, after 4 h of reflux. The resulting yellow (Bmpp-Sn) and pale-yellow (Ampp-Sn) solids were filtered, washed with methanol, and dried at room temperature. They were recrystallized from methanol and stored over fused CaCl_2_. Suitable thread-like yellow single crystals of Bmpp-Sn for X-ray diffraction were grown at room temperature from slow evaporation of methanol solution after seven days.

#### 2.2.1. 4-Acetyl-3-methyl-1-phenyl-2-pyrazolin-5-one Sulfanilamide: Ampp-Sn (**1**)

Pale yellow solid, yield 74%, mp 232−234°C; IR KBr (*ν*_max_, cm^−1^): 3496 (N–H), 2954 (C–H), 1633 (C=N), 1503 (C=O). ^1^H NMR (600 MHz, DMSO): *δ* (ppm) 13.1 (s, 1H, –NH), 8.0 (s, 1H, C=N–H), 7.9–7.2 (m, 9H, aromatic–H), 2.4 (s, 3H, CH_3_). ^13^C NMR (600 MHz, DMSO): *δ* (ppm) 165.4 (s, C=O), 164.5 (s, C=N), 148.2 (s, C–S) 142.8–116.6 (s, aromatic carbons), 17.6 (s, pyrazolone CH_3_), 17.5 (s, acetyl CH_3_); APCI-MS:* m*/*z* 371.11 ([M + H]^+^, 100%). Anal. Calcd. C_18_H_18_N_4_O_3_S (370.11): C, 58.36; H, 4.90; N, 15.13%. Found: C, 58.42; H, 4.88; N, 15.18%.

#### 2.2.2. 4-Benzoyl-3-methyl-1-phenyl-2-pyrazolin-5-one Sulfanilamide: Bmpp-Sn (**2**)

Yellow solid, yield 69%, mp 102−104°C; IR KBr (*ν*_max_, cm^−1^): 3497 (N–H), 2927 (C–H), 1639 (C=N), 1504 (C=O). ^1^H NMR (600 MHz, DMSO): *δ* (ppm) 12.8 (br, 1H, –NH), 8.0 (s, 1H, C=N–H), 7.8–7.1 (m, 14H, aromatic–H), 2.2 (s, 3H, CH_3_). ^13^C NMR (600 MHz, DMSO): *δ* (ppm) 190.5 (s, C=O), 139.3 (s, C=N), 148.2 (s, C–S) 132.2–110.7 (s, aromatic carbons), 16.5 (s, pyrazolone CH_3_); APCI-MS:* m*/*z* 433.13 ([M + H]^+^, 100%). Anal. Calcd. C_23_H_20_N_4_O_3_S (432.13): C, 63.87; H, 4.66; N, 12.96%. Found: C, 63.84; H, 4.67; N, 12.79%.

### 2.3. General Procedure for the Synthesis of Acylpyrazolone Schiff Bases Metal Complexes

To a solution of Bmpp-Sn (2 mmol, 0.87 g) and Ampp-Sn (2 mmol, 0.74 g) in hot ethanol (40 mL) each in a separate round bottom flask aqueous solution of 2 mmol of corresponding metal salts was added in drops while stirring under reflux and followed by the addition of NaOH (2 mmol, 0.08 g) to precipitate the metal complex. After 4 h of reflux, the precipitates in different colours were filtered, washed with ethanol/water (1 : 1), dried at room temperature, and stored over fused CaCl_2_.

#### 2.3.1. Bis(4-acetyl-3-methyl-1-phenyl-2-pyrazolin-5-one sulfanilamide)diaquamanganese(II) Monohydrate Mn(Ampp-Sn)_2_(H_2_O)_2_·H_2_O (**1a**)

Yellow solid; yield 81%; mp 210−212°C; Molar cond. (10^−3^ M in DMF): 14.40 ohm^−1^ cm^2^ mol^−1^; *μ*_eff_ (B.M.): 5.61; UV-Vis (DMF) *λ*_max_ nm: 259 (*π* → *π* ^*∗*^), 377 (n → *π* ^*∗*^), 431 (d→d); IR KBr (*ν*_max_, cm^−1^): 3484 (N–H), 3392 (O–H), 2920 (C–H), 1624 (C=N), 824 (H_2_O), 634 (M–N), 488 (M–O); Anal. Calcd. C_36_H_40_N_8_O_9_S_2_Mn (847.17): C 50.99, H 4.76, N 13.22%, Found: C 51.01, H 4.58, N 13.21%.

#### 2.3.2. Bis(4-acetyl-3-methyl-1-phenyl-2-pyrazolin-5-one sulfanilamide)diaquacobalt(II) Co(Ampp-Sn)_2_(H_2_O)_2_ (**1b**)

Yellow solid; yield 82%; mp 150−151°C; Molar cond. (10^−3^ M in DMF): 11.22 ohm^−1^ cm^2^ mol^−1^; *μ*_eff_ (B.M.): 4.43; UV-Vis (DMF) *λ*_max_ nm: 274 (*π* → *π* ^*∗*^), 377 (n → *π* ^*∗*^), 408, 632, 699 (d→d); IR KBr (*ν*_max_, cm^−1^): 3491 (N–H), 3391 (O–H), 2923 (C–H), 1619 (C=N), 831 (H_2_O), 631 (M–N), 486 (M–O); Anal. Calcd. C_36_H_38_N_8_O_8_S_2_Co (833.16): C 51.85, H 4.60, N 13.45%, Found: C 51.61, H 4.51, N 13.11%.

#### 2.3.3. Bis(4-acetyl-3-methyl-1-phenyl-2-pyrazolin-5-one sulfanilamide)diaquanickel(II) Monohydrate Ni(Ampp-Sn)_2_(H_2_O)_2_·H_2_O (**1c**)

Yellow solid; yield 79%; mp 157−158°C; Molar cond. (10^−3^ M in DMF): 16.02 ohm^−1^ cm^2^ mol^−1^; *μ*_eff_ (B.M.): 2.93; UV-Vis (DMF) *λ*_max_ nm: 257 (*π* → *π* ^*∗*^), 324 (n → *π* ^*∗*^), 596, 674 (d→d); IR KBr (*ν*_max_, cm^−1^): 3488 (N–H), 3398 (O–H), 2934 (C–H), 1620 (C=N), 823 (H_2_O), 622 (M–N), 488 (M–O); Anal. Calcd. C_36_H_40_N_8_O_9_S_2_Ni (850.93): C 50.77, H 4.74, N 13.16%, Found: C 50.65, H 4.71, N 13.30%.

#### 2.3.4. Bis(4-acetyl-3-methyl-1-phenyl-2-pyrazolin-5-one sulfanilamide)diaquacopper(II) Monohydrate Cu(Ampp-Sn)_2_(H_2_O)_2_·H_2_O (**1d**)

Yellow solid; yield 83%; mp 298−299°C; Molar cond. (10^−3^ M in DMF): 7.04 ohm^−1^ cm^2^ mol^−1^; *μ*_eff_ (B.M.): 1.90; UV-Vis (DMF) *λ*_max_ nm: 261 (*π* → *π* ^*∗*^), 365 (n → *π* ^*∗*^), 618, (d→d); IR KBr (*ν*_max_, cm^−1^): 3482 (N–H), 3392 (O–H), 2922 (C–H), 1617 (C=N), 850 (H_2_O), 628 (M–N), 498 (M–O); Anal. Calcd. C_36_H_40_N_8_O_9_S_2_Cu (855.78): C 50.48, H 4.71, N 13.09%, Found: C 50.44, H 4.59, N 13.21%.

#### 2.3.5. Bis(4-benzoyl-3-methyl-1-phenyl-2-pyrazolin-5-one sulfanilamide)diaquamanganese(II) Mn(Bmpp-Sn)_2_(H_2_O)_2_ (**2a**)

Pale yellow solid; yield 82%; mp 180−182°C; Molar cond. (10^−3^ M in DMF): 8.19 ohm^−1^ cm^2^ mol^−1^; *μ*_eff_ (B.M.): 5.82; UV-Vis (DMF) *λ*_max_ nm: 277 (*π* → *π* ^*∗*^), 378 (n → *π* ^*∗*^), 424, 589 (d→d); IR KBr (*ν*_max_, cm^−1^): 3483 (N–H), 3398 (O–H), 2938 (C–H), 1623 (C=N), 844 (H_2_O), 619 (M–N), 470 (M–O); Anal. Calcd. C_46_H_42_N_8_O_8_S_2_Mn (953.19): C 57.91, H 4.44, N 11.75%, Found: C 57.72, H 4.14, N 11.22%.

#### 2.3.6. Bis(4-benzoyl-3-methyl-1-phenyl-2-pyrazolin-5-one sulfanilamide)diaquacobalt(II) Monohydrate Co(Bmpp-Sn)_2_(H_2_O)_2_·H_2_O (**2b**)

Pink solid; yield 89%; mp 144−146°C; Molar cond. (10^−3^ M in DMF): 10.30 ohm^−1^ cm^2^ mol^−1^; *μ*_eff_ (B.M.): 4.46; UV-Vis (DMF) *λ*_max_ nm: 262 (*π* → *π* ^*∗*^), 376 (n → *π* ^*∗*^), 408, 540 (d→d); IR KBr (*ν*_max_, cm^−1^): 3492 (N–H), 3398 (O–H), 2925 (C–H), 1618 (C=N), 849 (H_2_O), 624 (M–N), 489 (M–O); Anal. Calcd. C_46_H_44_N_8_O_9_S_2_Co (975.20): C 56.60, H 4.55, N 11.49%, Found: C 56.33, H 4.92, N 11.16%.

#### 2.3.7. Bis(4-benzoyl-3-methyl-1-phenyl-2-pyrazolin-5-one sulfanilamide)diaquanickel(II) Monohydrate Ni(Bmpp-Sn)_2_(H_2_O)_2_·H_2_O (**2c**)

Light green solid; yield 85%; mp 190−192°C; Molar cond. (10^−3^ M in DMF): 11.62 ohm^−1^ cm^2^ mol^−1^; *μ*_eff_ (B.M.): 2.89; UV-Vis (DMF) *λ*_max_ nm: 278 (*π* → *π* ^*∗*^), 377 (n → *π* ^*∗*^), 410, 683 (d→d); IR KBr (*ν*_max_, cm^−1^): 3489 (N–H), 3390 (O–H), 2935 (C–H), 1619 (C=N), 849 (H_2_O), 625 (M–N), 486 (M–O); Anal. Calcd. C_46_H_44_N_8_O_9_S_2_Ni (974.96): C 56.62, H 4.55, N 11.49%, Found: C 57.01, H 4.29, N 11.30%.

#### 2.3.8. Bis(4-benzoyl-3-methyl-1-phenyl-2-pyrazolin-5-one sulfanilamide)diaquacopper(II) Cu(Bmpp-Sn)_2_(H_2_O)_2_ (**2d**)

Brown solid; yield 92%; mp 238−240°C; Molar cond. (10^−3^ M in DMF): 6.41 ohm^−1^ cm^2^ mol^−1^; *μ*_eff_ (B.M.): 1.93; UV-Vis (DMF) *λ*_max_ nm: 273 (*π* → *π* ^*∗*^), 378 (n → *π* ^*∗*^), 442, 751 (d→d); IR KBr (*ν*_max_, cm^−1^): 3498 (N–H), 3399 (O–H), 2935 (C–H), 1617 (C=N), 840 (H_2_O), 623 (M–N), 484 (M–O); Anal. Calcd. C_46_H_42_N_8_O_8_S_2_Cu (961.80): C 57.39, H 4.40, N 11.65%, Found: C 57.19, H 4.31, N 11.32%.

### 2.4. X-Ray Diffraction Study of Bmpp-Sn (**2**)

Single crystal X-ray diffraction studies were performed at 200 K using a Bruker Kappa Apex II diffractometer with graphite monochromator, Mo K*α* radiation (*λ* = 0.71073 Å). APEXII was used for data collection and SAINT for cell refinement and data reduction. The structure was solved by direct methods using SHELXS-97 and refined by least-squares procedures using SHELXL-97 [21] with SHELXLE [22] as a graphical interface. Platon [23] and Ortep-3 [24] were used to prepare material and diagrams, respectively.

### 2.5. Antibacterial Studies

*In vitro* antibacterial activity of synthesized compounds was screened against Gram positive* Staphylococcus aureus* and* Bacillus pumilus* and Gram negative* Proteus vulgaris* and* Aeromonas hydrophila* bacterial isolates at 40 mg/mL in DMSO using the Kirby-Bauer disc diffusion technique based on the size of inhibition zone formed around the paper discs with some modifications [25]. Bacterial isolates were grown in freshly prepared nutrient broth growth media to obtain a minimum of 0.1 optical density OD of bacterial isolates suitable for screening. To a solidified about 20 mL Mueller-Hinton agar in Petri plates, 0.1 mL of test bacteria was spread over the medium using a sterilized spreader. Presterilized filter paper discs having a diameter of 6 mm which were impregnated into prepared solutions of synthesized ligands and complexes were placed in the inoculated Petri plates ensuring a reasonable equidistance from each other. Filter paper disc treated with DMSO was used as control while antibacterial chloramphenicol served as a standard drug and was used as a reference to evaluate the potency of the tested compounds under the same conditions. The Petri dishes were left for a few hours in the refrigerator for prediffusion and finally transferred to an incubator at 37°C for 24 h. This procedure was performed in triplicate. The bactericidal activities were determined by measuring the diameter of the zones showing complete inhibition of bacterial growth in millimeters and subtracting the diameter of the filter paper disc and finally dividing by 2 to obtain the exact zone of inhibition. The values were calculated as mean of triplicates.

### 2.6. Antioxidant (Free Radical Scavenging) Activity

Schiff base free radical scavenging activity and that of its metal complexes were tested against the stable free radical of 1, 1-diphenyl-2-picrylhydrazyl (DPPH), employing the method of Blois [26] with few modifications. A 0.1 mmol solution of the DPPH in methanol was prepared, and 1 mL of this solution was added to 3 mL prepared solutions of the test compounds in a mixture of DMSO and methanol in a mole ratio of 1 : 9, respectively, at different concentrations (0.13, 0.25, and 0.50 mg/mL). The same procedure was carried out for standard drug, ascorbic acid, and an equal volume of dissolving solvents as control. The mixture was shaken vigorously and allowed to stand at room temperature in the dark for 30 min. With the use of a spectrophotometer at a wavelength of 517 nm the absorbance was measured. The capability of synthesized compounds to scavenge DPPH radical was calculated using the expression

(1) $$\begin{array}{c} Scavenging\;\;activity\left(\;\%\;\right)=\frac{A_{0}-A_{1}}{A_{0}}\times 100, \end{array}$$

where *A*_0_ is the absorbance with control sample and *A*_1_ is the absorbance with test samples including that of standard drug.

## 3. Results and Discussion

### 3.1. Synthesis

**Scheme 1:**
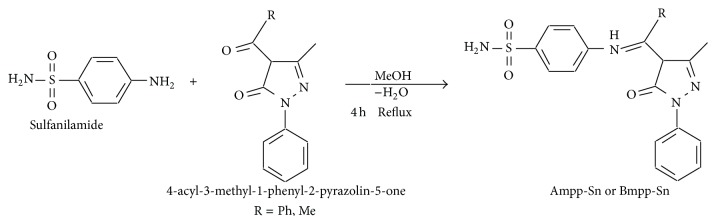
Synthesis of 4-acyl-3-methyl-1-phenyl-2-pyrazolin-5-one-sulfanilamide.

The acylpyrazolone Schiff base precursors, 4-acetyl-3-methyl-1-phenyl-2-pyrazolin-5-one and 4-benzoyl-3-methyl-1-phenyl-2-pyrazolin-5-one, were synthesized using the reported method [20], while the Schiff base ligands 4-acetyl-3-methyl-1-phenyl-2-pyrazolin-5-one sulfanilamide (Ampp-Sn)** 1** and 4-benzoyl-3-methyl-1-phenyl-2-pyrazolin-5-one sulfanilamide (Bmpp-Sn)** 2 **were afforded by the condensation reaction of an aromatic amine 4-aminobenzenesulfonamide (sulfanilamide), with 4-acetyl or 4-benzoyl-3-methyl-1-phenyl-2-pyrazolin-5-one in a 1 : 1 Molar ratio according to the synthetic Scheme 1.

They were obtained in more than 50% yield and recrystallized in methanol. Single threadlike crystal structure of** 2** distorted with a water molecule was obtained from slow evaporation of a solution of methanol. Treatment of** 1** or** 2** with an appropriate metal salt afforded the metal complexes** 1a**–**d** and** 2a**–**d** having a general scheme presented in (2), which shows synthesis of 4-acyl-3-methyl-1-phenyl-2-pyrazolin-5-one-sulfanilamide transition metal complexes. Their Molar conductance values infer a nonelectrolyte behavior in DMF [27]:

(2) MX2·nH2O→EtOH/H2Oreflux,4 hML2H2O2·nH2OM=Mn2+a,  Co2+b,  Ni2+c,  Cu2+d;  X=Cl−  or  CH3COO−;  L=Ampp-Sn1  or  Bmpp-Sn2.

The elemental percentage composition of** 1**,** 2**,** 1a**–**d,** and** 2a**–**d** found was in agreement with calculated values. Octahedral metal complexes with the corresponding Schiff base binding bidentately are proposed, having two molecules each of the Schiff base and two molecules of water to complete its octahedral geometry [28].

### 3.2. ^1^H and ^13^C NMR Spectroscopy

**Figure 1:**
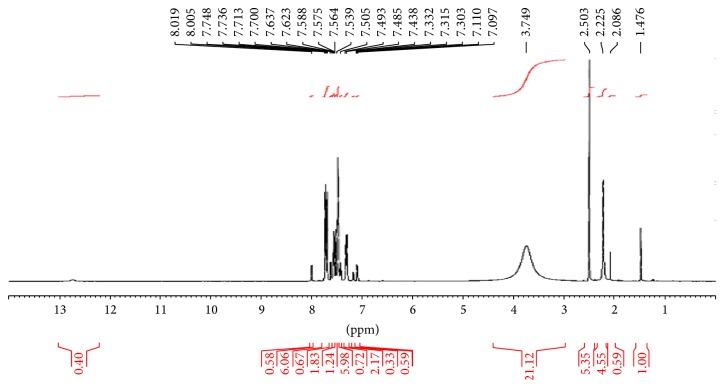
^1^H NMR spectrum of Bmpp-Sn (**2**).

The ^1^H NMR spectra of** 2** showed a multiplet at 7.8–7.1 ppm due to the aromatic protons and a singlet each at 12.8 ppm and at 8.0 ppm, Figure 1, assigned, respectively, to the sulfanilamide NH_2_ hydrogen –NH and the azomethine nitrogen H–N=C proton. The resonance peak due to methyl protons on the pyrazolone ring is displayed upfield at 2.2 ppm integrating for an unequivalent number of hydrogen atoms.

**Figure 2:**
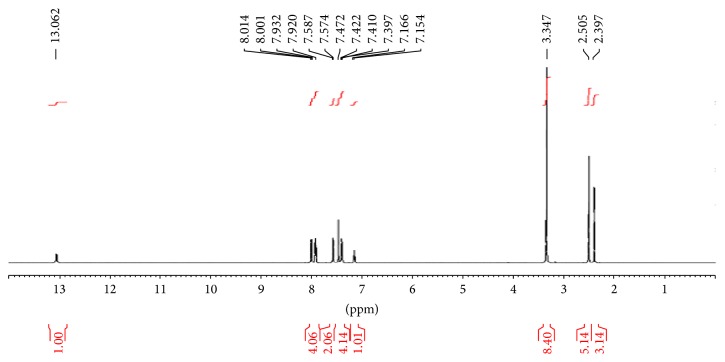
^1^H NMR spectrum of Ampp-Sn (**1**).

^1^H NMR spectrum of** 1** was inconclusive as resonance peaks were not resolved enough to assign all necessary chemical shifts; however, the peak on the far downfield is due to sulfanilamide NH_2_ hydrogen –NH at 13.1 ppm integrated for one proton. The two sharp peaks observed at the far upfield are due to the methyl proton groups, pyrazolone methyl at 2.5 ppm, and the acetyl methyl at 2.4 ppm, although integrated for a higher proton value. The signals at 7.9–7.2 ppm are assigned to the aromatic protons while the peak at 8.0 ppm is due to azomethine hydrogen H–N=C, Figure 2.

**Figure 3:**
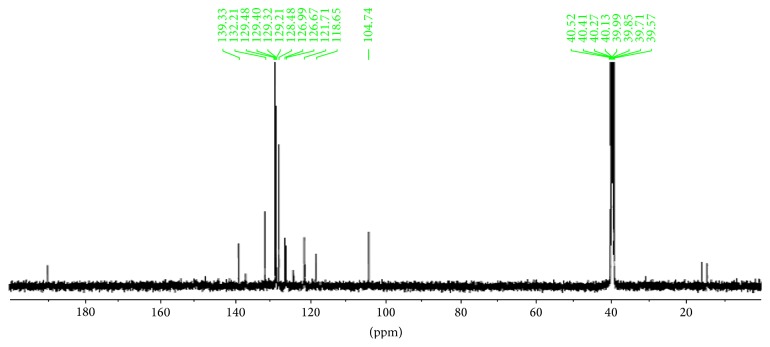
^13^C NMR spectrum of Bmpp-Sn (**2**).

**Figure 4:**
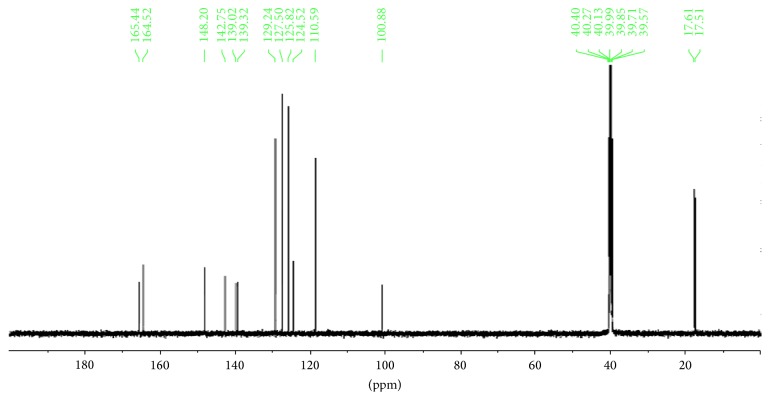
^13^C NMR spectrum of Ampp-Sn (**1**).

The ^13^C NMR spectra gave more information on the Schiff base ligands, Figures 3 and 4. A signal at 190.5 ppm in** 2** and that at 165.4 ppm in** 1** may be assigned to the carbonyl carbon C=O. The azomethine carbon is observed at 139.3 ppm in the spectrum of** 2** and at 164.5 ppm in** 1** [29]. Signals due to aromatic carbon atoms are resonating at a chemical shift of 132.2–110.7 ppm in** 2** and 142.8–116.6 ppm in** 1**. Aliphatic methyl carbon may be assigned to a signal at around 16.5 ppm in** 2** and the two methyl groups in** 1** are assigned to the signals at 17.6 and 17.5 ppm.

The resonance signal due to C–S may be assigned to the signal at 148.2 ppm in** 1**. Unfortunately, this particular signal could not be accounted for in** 4** but its mass spectrum and crystal X-ray structure do confirm the proposed molecular structure.

### 3.3. Mass Spectroscopy

**Figure 5:**
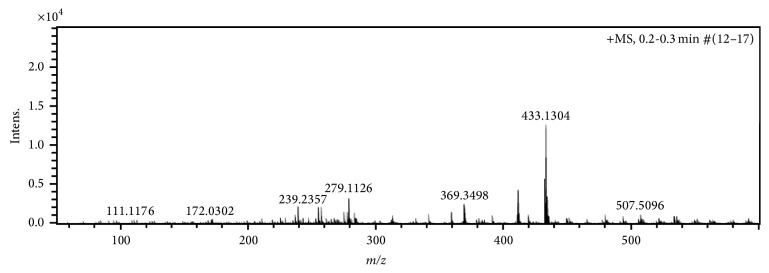
Mass spectrum of Bmpp-Sn (**2**).

**Figure 6:**
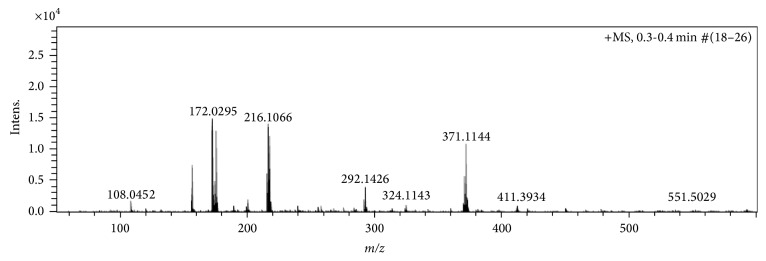
Mass spectrum of Ampp-Sn (**1**).

The molecular ion peak of the APCI source mass spectrum of** 2** and** 1** was observed at* m*/*z* 433 and* m*/*z* 371, respectively, and in agreement with the calculated theoretical Molar mass plus one hydrogen [M + H]^+^, equivalent protonated ligands, Figures 5 and 6.

Also the protonated benzoyl Schiff base precursor fragmentation peak was observed at* m*/*z* 279, that is, C_17_H_14_N_2_O_2_ in** 2**, while that of acetyl Schiff base precursor C_12_H_11_N_2_O_2_ was seen at* m*/*z* 216 in the spectrum of** 1**.

### 3.4. FT-IR Spectroscopy

The successful coordination of the sulfanilamide with acylpyrazolone to form the keto-imine tautomer Schiff bases was evident in the presence of the azomethine (C=N) and ketone carbonyl (C=O) vibrating bands in their IR spectra. The (C=N) was observed at 1639 cm^−1^ in** 2** and 1633 cm^−1^ in** 1** which was modified and appeared at a lower wave number region of 1627–1617 cm^−1^ in the IR spectra of the metal complexes [30]. Ketone carbonyl group (C=O) was prominently observed at 1504 cm^−1^ in** 2** and 1503 cm^−1^ in** 1**, which were reduced in peak size or have disappeared in their corresponding metal complexes spectra showing a transformation of the double bond of carbonyl into a metal oxygen bond –C–O–M [31]. The octahedral Schiff base metal complexes were completed with two water molecules each and this bond can be seen from the formation of a vibrating band at around 850–823 cm^−1^ in addition to the broad band at 3498–3390 cm^−1^ due to the –OH from the water molecules, which appeared originally at 3496–3497 cm^−1^ in** 1** and** 2** due to the –NH and –OH stretching frequencies [32]. The stretching bands that appeared at 634–619 cm^−1^ and 498–470 cm^−1^ are due to metal nitrogen bond M–N and that of metal oxygen M–O, respectively [33].

### 3.5. UV-Visible Spectroscopy and Magnetic Moment

**Figure 7:**
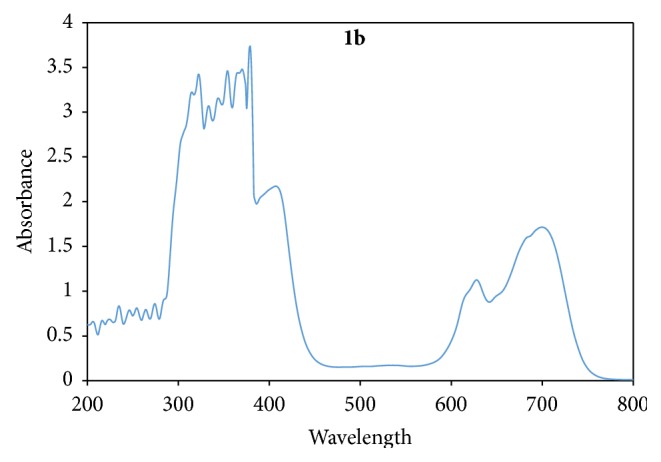
Electronic spectrum of Co(Ampp-Sn)_2_(H_2_O)_2_ (**1b**).

Bmpp-Sn and Ampp-Sn exhibited bands at the UV region due to charge transfer *π* → *π* ^*∗*^ and n → *π* ^*∗*^ transitions, which are usually intense as a result of the organic conjugate *π* bonds of the benzene ring [34]. Charge transfer transitions may interfere with each other, therefore preventing the expected transitions in the visible region of the electronic spectra of compounds to be observed, as seen in the spectra of some of the metal complexes reported herein [35]. Absorption bands and wavelength values carefully assigned are presented in the experimental section.** 2a** shows two bands in the visible region at 424 nm and 589 nm assigned to ^6^A_1g_ → ^4^T_2g_ and ^6^A_1g_ → ^4^T_1g_ transitions, respectively, corresponding to a d^5^ octahedral Mn(II) complex [36]. One band at 431 nm is assigned to ^6^A_1g_ → ^4^T_2g_ in** 1a**. The high spin d^5^ octahedral manganese complexes were corroborated with the magnetic moment values of 5.82 and 5.61 BM for** 2a** and** 1a,** respectively [37].** 2b** displays two bands in the visible region at 408 nm and 540 nm corresponding to *ν*_1_ (^4^T_2g_ → ^4^T_1g_) and (^4^A_2g_ → ^4^T_1g_) transitions, respectively. It similarly has a magnetic moment value of 4.46 BM, expected for an octahedral Co(II). Three bands were observed in the electronic spectra of** 1b** with magnetic moment of 4.43 BM, at 408 nm, 632 nm, and 699 nm assigned as (^4^T_2g_ → ^4^T_1g_), (^4^A_2g_ → ^4^T_1g_), and (^4^T_1g(p)_ → ^4^T_1g_) transitions, respectively, peculiar to a d^7^ octahedral geometry [38] (Figure 7).

**Figure 8:**
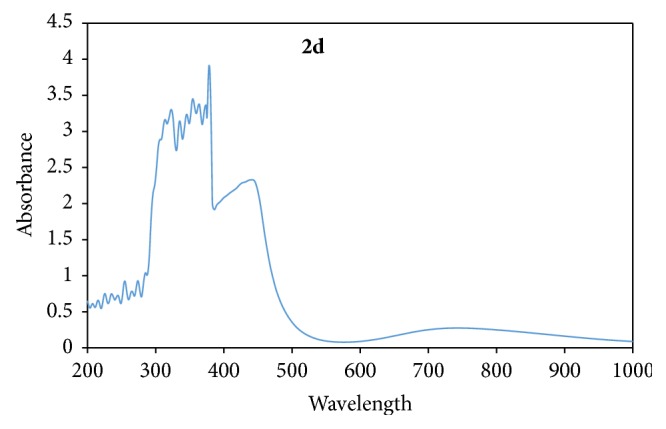
Electronic spectrum of Cu(Bmpp-Sn)_2_(H_2_O)_2_ (**2d**).

The absorption bands at 410 nm and 683 nm were assigned to ^3^A_2g_ → ^3^T_1g(p)_ and ^3^A_2g_ → ^3^T_1g(F)_, respectively, in** 2c**. In** 1c** the d-d bands at 596 nm and 674 nm are assigned to ^3^A_2g_ → ^3^T_1g(F)_ and ^3^A_2g_ → ^3^T_2g(F)_ transitions, respectively. These transitions correspond to a d^8^ Ni(II) with octahedral geometry. Their magnetic moment of 2.89 and 2.93 BM for** 2c** and** 1c**, respectively, further justifies the proposed geometries [39]. Two bands in the electronic spectra of** 2d**, observed as a strong band at 442 nm and a broad band that peaks at 751 nm, were assigned to ^2^B_1g_ → ^2^E_g_ and ^2^B_1g_ → ^2^B_2g_ transitions, respectively (Figure 8), with a magnetic moment value of 1.93 BM.** 1d** exhibited one single broad band characterized by a distorted octahedral Cu(II) at 618 nm which is assigned to ^2^B_1g_ → ^2^B_2g_ transition, with a magnetic moment value of 1.90 BM [38].

### 3.6. Thermogravimetric Analysis

**Figure 9:**
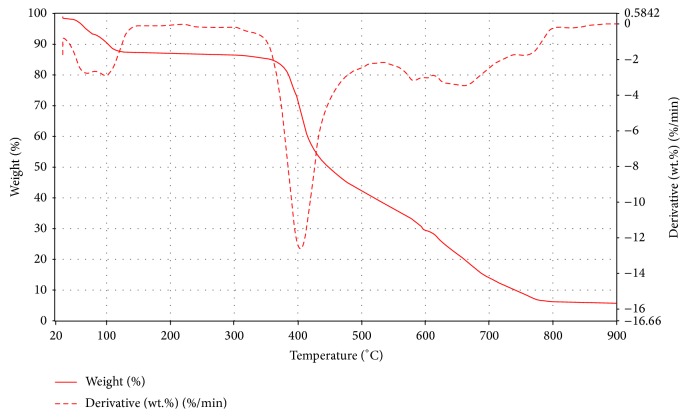
TGA and DTG curve of Co(Bmpp-Sn)_2_(H_2_O)_2_·H_2_O (**2b**).

TGA in collaboration with DTG results shows a multistep decomposition pattern in the thermograms of some of the metal complexes under investigation. Generally, relatively low thermal decomposition mass losses observed below 300°C are due to the removal of the coordinated and noncoordinated water molecules, although not so in some of the metal complexes with coordinated water molecule [40]. As they exhibited multiple decomposition steps, their assignments were not unequivocal. In** 2a** the decomposition due to the removal of the four water molecules is observed at 220°C. The major decomposition at around 550°C with a mass loss of about 44% may be attributed to the removal of a molecule of** 2** calculated as 45.3%. This decomposition was followed by a second, over a wide temperature range extending beyond 900°C, which may be attributed to the second Schiff base ligand, leaving a residue of manganese oxide. Schiff base ligands with more aromatic groups are generally more stable; hence the varying trends of decomposition are shown by** 1a**–**d **as we proceed. The decomposition due to the elimination of the water molecules in** 1a** is observed at 150 and 290°C. It was followed by a major decomposition at around 450°C attributed to the decomposition of a molecule of** 1** with a mass loss of 40% and calculated as 43.7%. The final decomposition due to the second ligand extends beyond 900°C leaving the metal oxide. The removal of water molecules from** 4b** was observed at around 100°C. The major decomposition at 400°C was attributed to the loss of** 4** with a mass percentage of 45% which was calculated as 44.3%. The third decomposition over a wide temperature range was attributed to the second Schiff base ligand leaving a cobalt oxide residue behind with a percentage mass of 6.9% and calculated as 7.7% (Figure 9). The thermogram for** 1b** exhibited multiple decompositions bringing about unequivocal assignments an evidence of its low thermal stability. However, the water molecules elimination can be observed at 160°C.

**Figure 10:**
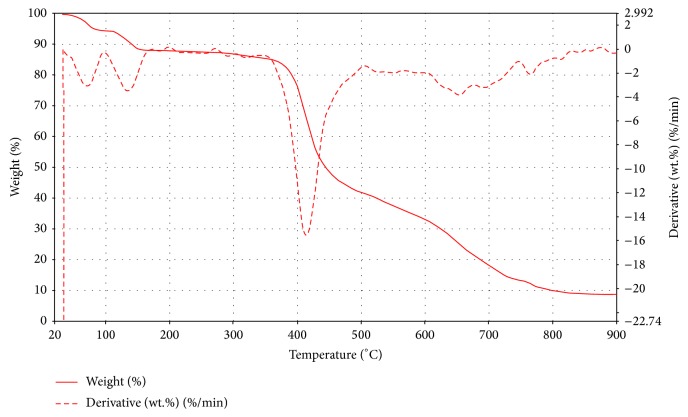
TGA and DTG curve of Ni(Bmpp-Sn)_2_(H_2_O)_2_·H_2_O (**2c**).

Complex** 2c** (Figure 10) exhibited decompositions due to water molecules at around 80 and 140°C. Its major decomposition occurred at 420°C associated with the removal of a molecule of** 2** with an equivalent mass loss of 50% and calculated as 50.9%. The final decomposition at around 810°C was attributed to the removal of a total of four molecules of water and two molecules of the Schiff base leaving behind a nickel oxide residue with a percentage mass of 9% and calculated as 8.7%.

**Figure 11:**
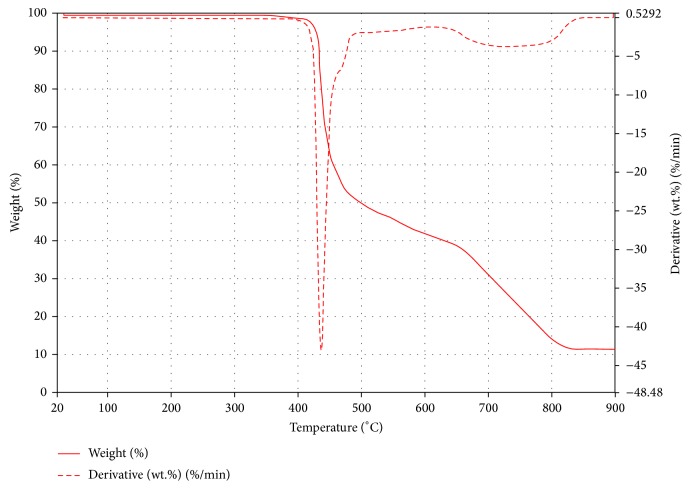
TGA and DTG curve of Cu(Ampp-Sn)_2_(H_2_O)_2_·H_2_O (**1d**).

Another multistep decomposition trend was observed in** 1c**. The elimination of the water molecules was evident from the decompositions at 170 and 280°C. A major decomposition due to the removal of a** 1** molecule was observed at 440°C with a mass loss of 44% which is in agreement with the calculated mass loss of 43.5%. The final decomposition due to the removal of the second Schiff base extends beyond 900°C which was terminated with the left over nickel oxide. The thermal decomposition of** 2d** showed an unexpected one decomposition step at 330°C which cannot be easily assigned. This observation may be due to the formation of gaseous reaction products. Also unexpectedly, the coordinated water molecule in** 1d** cannot be accounted for in the TGA curve but a major decomposition at 440°C may be due to the elimination of one Schiff base ligand with a mass loss of 45%, calculated as 43.2% (Figure 11). This was followed by a second and final decomposition attributed to the fragmentation of the last** 1** molecule, leaving behind a residue of the metal oxide with a remaining mass of around 11%, calculated as 8.8%.

**Figure 12:**
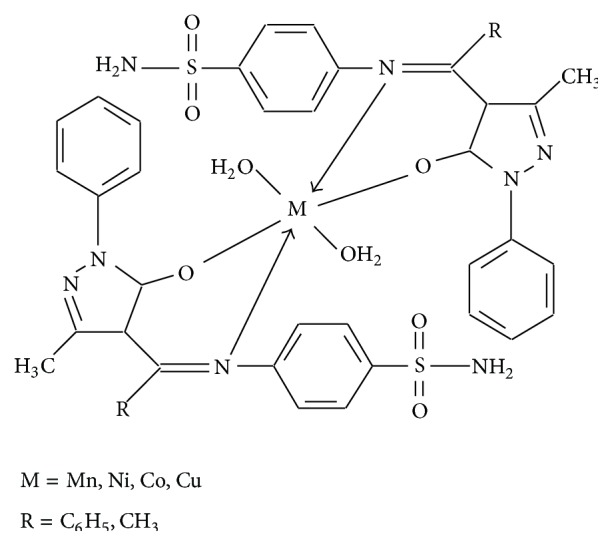
Proposed structural scheme for metal complexes.

On the basis of elemental analysis and spectroscopic and TGA results the proposed structures of metal complexes may be represented as seen in Figure 12.

### 3.7. Crystallographic Data

Slow evaporation of** 2** in methanol afforded needle-like single crystals of Schiff base** 2** with a water molecule distortion formulated as C_23_H_20_N_4_O_3_S·H_2_O. A summary of crystal data is presented in Table 1.

**Figure 13:**
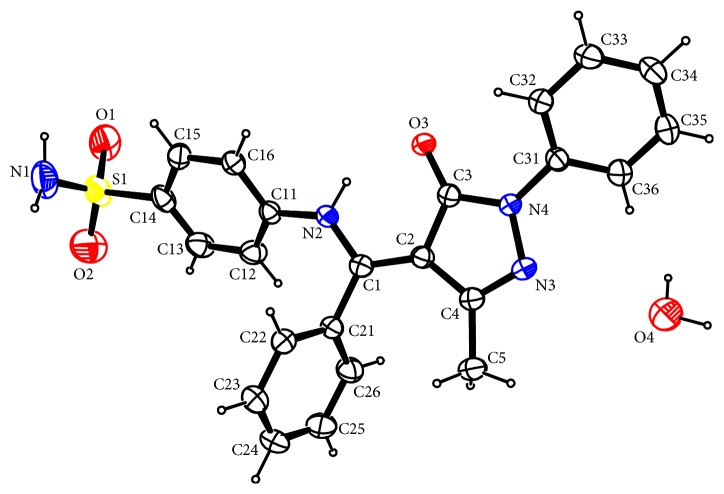
Molecular crystal structure of Bmpp-Sn·H_2_O.

Solid state molecular structure of Bmpp-Sn·H_2_O as seen in Figure 13 is essentially planar with the plane of the methylpyrazolone group but with the phenyl groups turned out of the plane in accordance with reported crystal structures [18, 28]. The phenyl rings C11–C16, C21–C26, and C31–C36 make dihedral angles of 39.44(12)°, 66.26(13)°, and 34.38(13)°, respectively, with the methylpyrazolone group.

**Figure 14:**
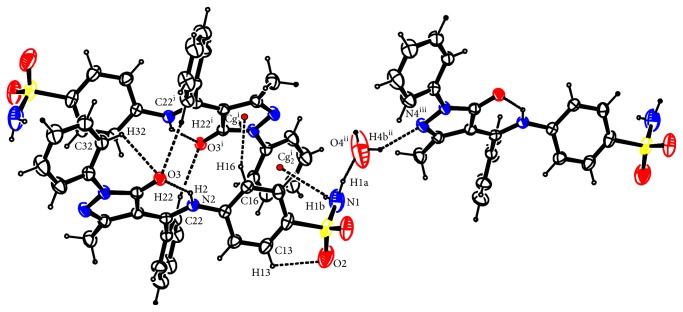
Hydrogen bonding in Bmpp-Sn·H_2_O.

There are three intramolecular bonds: one short N2⋯H2⋯O3 hydrogen bond of 1.90 Å and two long C13⋯H13⋯O2 and C32⋯H32⋯O3 hydrogen bonds of 2.48 Å each (Figure 14).

Pairs of Bmpp-Sn·H_2_O are alternatively stacked in the c-axis direction and connected with two C22⋯H22⋯O3 hydrogen bonds of 2.45 Å, two C16⋯H16⋯C (pyrazole ring) *π*-ring interactions of 2.66 Å, and two N1⋯H1B⋯C (C31–C36 phenyl ring) *π*-ring interaction of 2.70 Å. A water molecule connects adjacent Bmpp-Sn·H_2_O in the b-axis direction with two hydrogen bonds N1⋯H1A⋯O4⋯H4B⋯N4 of length 1.74 and 2.13 Å, respectively. Crystallographic data for the structural analysis have been deposited with the Cambridge Crystallographic Data Centre, CCDC, number 923953.

### 3.8. Antibacterial Activity

A generally moderate to poor activity of reported compounds with some showing no activity at all is observed relative to the standard chloramphenicol drug, as seen from the mean values of bacterial growth inhibition zones carried out in triplicate at 40 mg/mL concentration.** 2** exhibited a broad spectrum activity (active against all selected bacterial isolates) and showed the highest zone of inhibition value of 24 mm against* Aeromonas hydrophila *(Table 2).** 2b**,** 2c**,** 1c**, and** 1** on the other hand exhibited activity against three of the selected bacterial isolates.

Although it is expected that chelation of metal ion to Schiff base does increase the possibility of microbial growth inhibition [41], the observed variation herein may be as a result of impermeability of bacterial isolates cell wall or the nature of their ribosomes [42].

### 3.9. Antioxidant (Free Radical Scavenging) Activity

**Figure 15:**
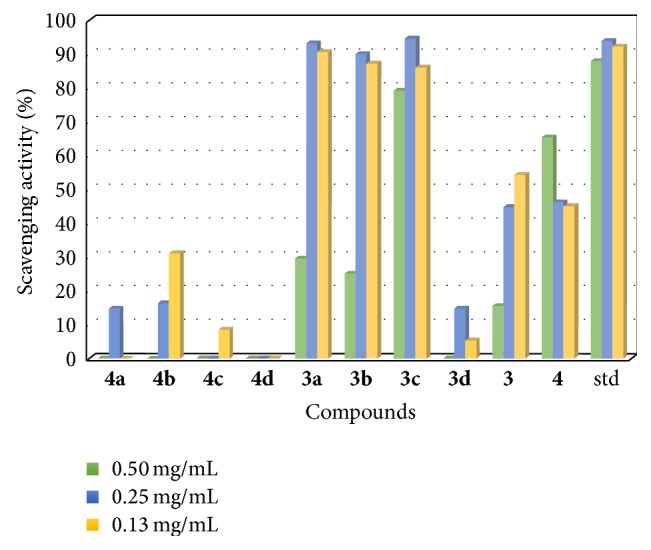
Chart showing the scavenging activity.

The reduction/scavenging capability is taken as the decrease in absorbance at a constant wavelength relative to the control sample which is made possible by antioxidant properties [43]. It is evident that the potential antioxidant properties of some naturally occurring ingredients from plants and synthetic analogs are as a result of having electron-donating groups [44] and as such the inductive effects of the sulfur and nitrogen groups in** 1** and** 2** may be able to push electron density towards the free radicals to produce relatively stable molecules. The metal complexes exhibited a generally low antioxidant property compared to standard drug ascorbic acid (Figure 15). However,** 1c** showed very strong antioxidant activities having values almost equal to that of ascorbic acid in all three different concentrations.** 1a** as well as** 1b** exhibited strong antioxidant properties higher than their Schiff base ligand** 1,** which is in agreement with previously reported compounds coordinated to transition metal ions [45].

On comparing the metal complexes with their corresponding ligands,** 2** showed a stronger free radical scavenging property at all three different concentrations contrary to** 1**.

## 4. Conclusions 

The reported results herein support the successful synthesis of new possible therapeutic Schiff bases and their metal complexes. A keto-imine tautomer of** 1** and** 2** has been established with good bioactivity, more of which were free radicals scavengers (antioxidant activity). Single crystal X-ray analysis further confirms the structure of** 2** and an octahedral geometry of metal complexes with two molecules of Schiff base ligand and H_2_O molecule each have been proposed by way of analytical and spectroscopic techniques. A good number of the synthesized compounds have been identified as potential bactericidals and putting their antioxidant activities into consideration, synthesized compounds may be useful antitumour candidates.

## Figures and Tables

**Table 1:** Crystal data for **2**.

Compound	Bmpp-Sn
Formula	C_23_H_20_N_4_O_3_S·H_2_O
Crystal colour and form	Orange/block
Formula weight	450.52
Crystal system	Monoclinic
Space group	C2/c
*a*	23.3366 (4) (Å)
*b*	14.7706 (3) (Å)
*c*	12.5787 (2) (Å)
*α*	90 (2)°
*β*	100.727 (2)°
*V*	4257.21 (13) (Å^3^)
*Z*	8
*D*_(calc)_	1.406 (Mg cm^−1^)
*F*(000)	1888
*θ* range	2.2–28.3 (°)
Crystal size	0.24 × 0.38 × 0.56 (mm)
Reflections measured	19945
Independent/observed	5285/4408
Mu(MoKa)	0.71073 (/mm)
Temperature	296 (K)
Parameters	108

**Table 2:** Zone of growth inhibition exhibited by sulfanilamide Schiff bases and metal complexes at 40 mg/mL (mm).

Ligand and complexes	*Staphylococcus aureus *	*Bacillus pumilus *	*Proteus vulgaris *	*Aeromonas hydrophila *
**2**	12.5	7.5	10.3	24.0
**1**	NI	5.0	8.5	4.0
**2a**	8.5	NI	NI	8.0
**2b**	13.3	16.0	NI	15.0
**2c**	8.0	NI	11.5	21.0
**2d**	NI	10.3	NI	8.0
**1a**	NI	NI	NI	8.5
**1b**	NI	9.0	NI	NI
**1c**	4.5	6.0	NI	6.0
**1d**	NI	NI	8.5	15.0
Chloramphenicol	30.0	20.0	42.0	40.0
DMSO	NI	NI	NI	NI

NI = no inhibition.

## Additional Information

Crystallographic data for 4-benzoyl-3-methyl-1-phenyl-2-pyrazolin-5-one sulfanilamide reported herein has been deposited with the Cambridge Crystallographic Data Centre (CCDC) number 923953. Copies of this information may be obtained free of charge from the Director, CCDC, 12 Union Road, Cambridge CB2 1EZ [Fax: +44 1223 336 033], or deposit@ccdc.cam.ac.uk or http://www.ccdc.cam.ac.uk.
